# Knowing your ABCs: Extending the assessment of stimulus-response (S-R) and cognitive-mediation (C-M) beliefs

**DOI:** 10.1371/journal.pone.0269928

**Published:** 2022-06-14

**Authors:** Martin J. Turner, Nanaki J. Chadha, Andrew G. Wood

**Affiliations:** Department of Psychology, Faculty of Heath, Psychology, and Social Care, Manchester Metropolitan University, Manchester, United Kingdom; University of Vienna Faculty of Psychology: Universitat Wien Fakultat fur Psychologie, AUSTRIA

## Abstract

Recently, researchers have proposed four superordinate emotion beliefs that supposedly influence emotion regulation and emotion reactivity. Two of these proposed emotion beliefs are captured in the cognitive mediation beliefs questionnaire (CMBQa), namely stimulus-response (S-R) generation beliefs and cognitive mediation (C-M) change beliefs. The remaining two proposed emotion beliefs, C-M generation beliefs and S-R change beliefs, are yet to be operationalised in psychometric form. It is important to validate measurement for all four emotion beliefs in order for them to be used in research and practice. The current paper reports the development and initial validity testing of the CMBQb (studies 1–3), which concerns only C-M generation beliefs and S-R change beliefs, and then tests the four-factor structure of the combined CMBQa (S-R generation, C-M change) and CMBQb (C-M generation, S-R change): the CMBQc (study 4). Some support was found for the four-factor structure of the CMBQc, with factor analyses revealing good fit to the data with a four-factor solution. Also, scores indicating greater C-M generation and change beliefs, and lower S-R generation and change beliefs, were related to more adaptive, and less maladaptive, emotion regulation tendencies. In addition, there was some evidence that greater C-M change beliefs, and lower S-R generation and change belief, were related to better affective and emotion reactivity outcomes. Implications of the CMBQc for research and practice are discussed within the context and emotion regulation science, and cognitive behavioural psychotherapy.

## Introduction

Attempting to influence the experience and expression of emotion is known in scientific literature as emotion regulation [1]. Successful emotion regulation can aid adaptive social, occupational, psychological health, and physical health [e.g., 2–4]. One prominent conceptualisation of emotion regulation is Gross’ [1] process model of emotion regulation, which offers five major emotion regulation strategies, namely situation selection, situation modification, attentional deployment, cognitive change, and response modulation. Of particular importance, is the emotion regulation strategy of cognitive reappraisal (or cognitive change), which is the modification of one’s appraisal of a situation in order to alter its emotional impact [5]. Cognitive reappraisal is demonstrably one of the most effective [6], and well-studied [7] emotion regulation strategies, associated with adaptive psychophysiological [8], and neurological [9] outcomes.

The notion that cognitive reappraisal is a highly effective emotion regulation strategy is in line with dominant psychotherapies in which cognitive mediation is a key axiomatic principle [10]. Specifically, second wave cognitive behavioural therapies (CBTs), such as rational emotive behaviour therapy (REBT [11]), hold that in the face of an adverse situation, our thoughts about the event largely determines our emotional reactions, and thus, to regulate our emotional reactions, one can modify one’s thoughts about the adverse situation [12]. The hypothesis that our thoughts mediate between the environment and our emotional responses is supported by theories of emotion [13], and scientific evidence (see [14], for a review). In second wave CBTs it is typical to help patients and clients to understand the important role of cognition in their emotions, and to encourage them to take charge of their cognitions in order to enable greater emotion regulation [15]. For example, within REBT [11], cognitive mediation is represented by the ABC framework whereby core beliefs (B) mediate between adverse events (A) and emotional consequences (C). In REBT, clients and patients are introduced to the notion that their cognitions about events have a considerable impact on their psychological wellbeing. Recognising that thoughts, and not events alone, lead to emotions, is known as B-C thinking, and in contrast, believing that event directly causes emotions despite deliberative cognition, is known as Adversity-Consequences thinking. Beliefs-Consequences thinking enables the individual to exercise some volition over their emotions by regulating their thoughts [16].

Given the importance of cognitive reappraisal in regulating one’s emotions, predetermining or predicting factors of cognitive reappraisal are worthy of investigation. In other words, we can ask the question, what factors make cognitive appraisals attempts more likely? One potential answer is “emotion beliefs” ([17], p. 74), which are considered to be beliefs about emotion and emotion regulation. Individuals differ in how they think about their emotions and how these emotions can be regulated, and these differences in beliefs are consequential for emotion regulation [18, 19]. Amidst a call for more empirical research in the area of emotion beliefs [20], in a recent study [21] conceptualised four emotion beliefs. The four emotion beliefs capture the aforementioned idea of cognitive mediation that is the centre of second wave CBTs, and cognitive reappraisal specifically. The four emotion beliefs are:

1. Stimulus-Response (S-R) generation beliefs (emotions are caused by events)
2. Stimulus-Response (S-R) change beliefs (changes in the situation lead to emotion change)
3. Cognitive Mediation (C-M) generation beliefs (emotions are cognitively mediated)
4. Cognitive Mediation (C-M) change beliefs (changes in cognition lead to emotion change).

Cleary, S-R beliefs reflect Adversity-Consequences thinking, whilst C-M beliefs reflect Beliefs-Consequences thinking, in CBT (i.e, REBT) terms. That is, S-R beliefs reflect the idea that emotion is dependent upon situational events, and C-M beliefs reflect the idea that emotion is dependent upon the one’s *cognitions about* situational events. S-R and C-M emotion beliefs are considered to be superordinate and dispositional beliefs, not entirely unlike the superordinate emotion beliefs of goodness and controllability proposed by Ford and Gross [17]. Goodness beliefs concern the extent to which emotions are good versus bad (including desirability and usefulness) and controllability beliefs concern the extent to which emotions are controllable versus uncontrollable. Goodness beliefs are thought to guide the trajectory of emotion regulation (i.e., what people want to feel), whilst controllability beliefs are thought to guide the occurrence of emotion regulation (i.e., whether regulation is initiated). Goodness beliefs are associated with emotional and wellbeing outcomes, such that those who believe emotions are bad have heightened negative emotional responses to stressors [22] and worse psychological health (e.g., [22, 23]). Controllability beliefs are linked to important emotional and social outcomes (e.g., [24–26]). For example, Tamir et al. [27] found that participants who believe that people can control their emotions were more effective in controlling their emotions and report enjoying long-term emotional and social benefits.

Of course, goodness and controllability are not the only beliefs individuals hold about emotions [17], and S-R and C-M beliefs are not antithetical to goodness and controllability beliefs. Rather, the S-R and C-M beliefs offered by Turner et al. [21] are supplementary to those of goodness and controllability. Further, the notion that changes in cognition lead to emotion change (C-M beliefs) might be somewhat dependent upon, or co-occurring with, beliefs about the controllability of emotion per se. Therefore, whilst S-R and C-M beliefs differ to goodness and controllability beliefs, different beliefs may interact to determine emotion regulation and reactivity outcomes.

One of the notable features of S-R and C-M beliefs is that two-factor view of emotion is captured (e.g., [28, 29]), whereby one factor concerns the generation of emotion, and another factor concerns emotional change. This two-factor approach is useful for analysing processes, individual differences, and fashioning clinical interventions [30]. The importance of separating out S-R and C-M beliefs across generation and change was borne by the findings of Turner et al. [21]. Specifically, Turner and colleagues developed a psychometric, the cognitive-mediation beliefs questionnaire (CMBQa), for the proposed four emotion beliefs, and found that S-R generation beliefs and C-M change beliefs formed reliable factors in the factor analyses, whilst C-M generation beliefs and S-R change beliefs did not. Follow-up data indicated that greater C-M change beliefs and lower S-R generation beliefs were related to higher cognitive reappraisal tendencies (adaptive emotion regulation), a greater ability to control thoughts, more positive mental health outcomes, and lower emotion reactivity.

Initial findings for the four proposed emotion beliefs are promising. However, because C-M generation and S-R change beliefs did not form reliable factors in the factor analyses by Turner et al. [21], research needs to ascertain the factorial validity and utility of C-M generation and S-R change beliefs. If indeed the four proposed emotion beliefs are valid ideas relevant to the study of emotions, researchers and practitioners should have valid and reliable questionnaires that assesses all four beliefs. This will enable a more comprehensive assessment of how these beliefs interact with each other, and also the extent to which they collectively and or discriminately influence emotion regulation and reactivity.

Therefore, the chief aim of the current paper is to validity test a new psychometric questionnaire pertaining to C-M generation and S-R change beliefs, the CMBQb, using items produced by Turner et al. [21] in the development of the CMBQa. To be clear, in the current study we aim to validity test a questionnaire (CMBQb) that pertains only to the two factors that did not emerge in Turner et al. [21]. To achieve this, three studies in the current paper report empirical efforts that align with psychometric questionnaire development guidelines for a latent variable approach [31, 32]. Study 1 reports exploratory factor analysis (EFA), study 2 reports confirmatory factor analysis (CFA), and study 3 reports additional CFA and criterion validity. Fourth and finally, in a further study (Study 4), we report the results of a four-factor CFA inclusive of both CMBQa (S-R generation, C-M change) and CMBQb (S-R change, C-M generation) factors. In Study 4 criterion validity tests are also undertaken to examine the relationships between the four CMBQ factors and variables concerning emotion regulation and emotion reactivity.

We pose the question: to what extent do individuals’ beliefs about the generation and regulation of their emotions influence their emotion reactivity? Initially, we focus on the proposed C-M generation and S-R change beliefs of the CMBQb.

## Study 1: Exploratory Factor Analysis (EFA)

For a detailed portrayal of the item generation process employed in the current study, see Turner et al. [21]. For the current study, we used items reflecting C-M generation and S-R change beliefs only (and no S-R generation and C-M change items), yielding a total of 24 suitable items for analyses. Following the psychometric questionnaire development guidelines (e.g., [32]), exploratory factor analysis (EFA) was used to assess the underlying two-factor structure of the 24-items [33]. We conducted EFA first, and then followed up with confirmatory factor analyses (CFA) with different samples [34]. We also recruited separate samples for each study in order to limit the attribution of results to cohort specific factors. For EFA the 24-items followed the stem, “When I experience an unpleasant or unwanted emotion, I believe that…”. Each item was scored against a five-point Likert-scale from 1 (*strongly disagree*) to 5 (*strongly agree*).

### Participants

An a priori participant item ratio of 10:1 was considered a suitable sample size for EFA, to minimize errors and maximize the accuracy and generalizability of population estimates [31, 35]. We aimed to recruit 240 participants via Prolific, which is an online research participant recruitment platform that has been successfully used in past research (e.g., [36]). Only those participants who were in full-time employment, were not currently students, and were based in the U.K were approached to take part in the study. A total of 252 respondents accessed the online survey platform Qualtrics. Data for two respondents were excluded due to poor data quality, specifically straight line responding (non-differentiation in ratings) which occurs when respondents lose their motivation to engage with a survey. Respondents were considered to be straight line responding if they selected the same Likert-scale point for an entire questionnaire. The final sample included 250 respondents (*M*age = 31.78, *SD*age = 7.90; female = 97; male = 153; Asian = 4; Black = 6; Mixed race = 5; White = 233; Other = 2). Ethical approval was granted from a University ethics committee, and informed written consent was gained from all participants.

### Data analysis

Data were screened for outliers (standardized z values > 3.29; [37]), and outliers were Winsorized (*n* = 13 from 6000 cases = .22%; [38]). Kaiser Meyer-Olkin (KMO = 87) measure and Bartlett’s test of sphericity, *X*^2^(276) = 2288.63, *p* < .001, indicated the suitability of the dataset for factor analysis. An EFA using Maximum Likelihood was carried out [39] using SPSS version 25. See S1 File for full (EFA) procedures.

## Results

In the EFA (see S2 File for the initial EFA) 9-items were deleted iteratively for failing to meet item retention criteria, and 15-items across the two factors were extracted accounting for 51.84% of the variance (Table 1). C-M generation (*n* = 7) and S-R change (*n* = 8) were negatively correlated (-.21). Although the factor loadings for items 7 (.595) and 21 (.599) were below .60, they were retained. We did not want to remove items that had loadings so close to the retention criteria, and these items can be further examined in CFA with a separate sample (study 2). EFA indicated the proposed two-factor approach to emotion regulation (e.g., [28]); C-M generation and S-R change.

**Table 1:** EFA outcomes for the two-factor model, with factor loadings, cross, loadings, and communalities.

Items	Factor-loading	Cross-loading S-R Change	Cross-loading C-M Generation	Communalities	% variance	Loading range	Loading mean	*α*	Eigen Value	*M*(SD)
Factor 1: C-M Generation					30.92	.60-.71	.66	.85	4.64	3.71(.76)
2	.710	.022	-	.472						
3	.687	.002	-	.473						
4	.677	.000	-	.472						
5	.663	-.003	-	.467						
1	.662	-.013	-	.439						
6	.640	-.008	-	.407						
7	.595	-.008	-	.354						
Factor 2: S-R Change					20.91	.60-.72	.67	.86	3.14	2.75(.96)
19	.724	-	-.048	.542						
17	.721	-	.049	.508						
20	.714	-	.104	.489						
16	.671	-	-.099	.488						
22	.641	-	-.044	.424						
18	.635	-	.005	.402						
24	.617	-	-.012	.384						
21	.599	-	.014	.356						

*Note*. Total variance explained = 51.84%; *χ*^2^ = 197.296, df = 76, *p* < .001

## Study 2: Confirmatory Factor Analysis (CFA)

A separate CFA sample was recruited to the EFA sample (e.g., [32]) to test the two-factor structure emergent from the EFA in study 1. To do this, we collected data using the 15-items derived from the EFA of study 1, which has two factors (C-M generation, and S-R change). Again, no S-R generation and C-M change (CMBQa) items were used. Therefore, the aim of the CFA in the current study was to confirm the proposed two-factor structure of the CMBQb.

### Participants

As in study 1, a priori participant:item ratio of 10:1 was considered a suitable sample size for EFA (e.g., [31]), but researchers suggest a CFA sample size of *n* = 500 to be very good [40]. Striking a balance between a 10:1 ration and *n* = 500, for the 15-items assessing the two factors, 331 respondents were recruited. We used the blocklist facility in Prolific to ensure that participants who had participated in the EFA study (study 1) could not be approached to take part in the CFA study (study 2). Of the 331 respondents, data for two respondents’ were excluded due to poor data quality (i.e., straight line responding). The final sample included 329 respondents (*M*age = 32.93; *SD*age = 8.73; female = 143; male = 186; Asian = 13; Black = 16; Mixed race = 4; White = 296). Ethical approval was granted from a University ethics committee, and informed written consent was gained from all participants.

### Data analysis

Participants completed the 15-item CMBQb on one occasion. Data were screened for outliers (standardized z values > 3.29), and outliers were Winsorized (*n* = 14 from 4,905 cases = .29%). Data were subjected to CFA using Structural Equation Modelling (SEM) in SPSS AMOS version 25 in which a two-factor model was tested using both correlated-factor and bifactor models (two CFAs in total; see S3 File for full CFA procedures). Bifactor models were tested to indicate whether the CMBQb assesses C-M generation and S-R change on a single factor, on two separate correlated factors, or both. Bifactor models allow the identification of a general factor in addition to multiple unique factors [41]. To compare the correlated two-factor model and the bifactor model, we inspected fit indices to determine which had the stronger model fit (S4 File) with Comparative Fit Index (CFI) differences of < .01 indicating no difference [42].

## Results

The 15-item correlated two-factor model was not an acceptable fit, *χ*^2^ = 285.909, df = 89, *p* < .001, RMSEA = .08 (90% CI = .072–.093), CFI = .89, NFI = .85, TLI = .87, SRMR = .06. After the removal of two items with low factor loadings (e.g., [43]), the correlated two-factor model was an acceptable fit, *χ*^2^ = 114.006, df = 62, *p* < .001, RMSEA = .05 (90% CI = .036–.065), CFI = .97, NFI = .93, TLI = .96, SRMR = .05. The acceptable two-factor model was taken forward to bifactor analyses.

The two-factor bifactor model was an acceptable fit, *χ*^2^ = 133.723, df = 52, *p* < .001, RMSEA = .07 (90% CI = .055–.084), CFI = .95, NFI = .92, TLI = .92, SRMR = .04. Although a good model fit was revealed, bifactor measures indicated that the CMBQb is not unidimensional and should not be assessed using a general factor (ΔCFI = .02). Specifically, for the bifactor model, PUC was .54, general ECV was .26, OmegaH was .22, and Relative Omega indicated that the general factor explained 24% of the variance. Additionally, all but one item had IECV coefficients below .50, indicating that items had stronger ties with their specific factors than the general factor [44]. Finally, the ARMPB for the factor loadings was 4.23% which is not acceptable [45], especially taking into account the other indices. In all, the ancillary bifactor measures for the two-factor models support the conceptualization of the CMBQb as multidimensional. Also, CFA indicated that a two-factor correlated factors model comprising 13 items (C-M generation = 7 items, S-R change = 6 items) produced the best fit to the data. For fit indices comparisons see S4 File, and for correlated and bifactor two-factor item loadings, Relative Parameter Bias, and IECV see S5 File.

## Study 3: Second CFA, and criterion validity

The purpose of study 2 was to test and confirm the two-factor structure of the CMBQb using CFA with a separate sample of adults. The CFA supported a correlated two-factor structure, comprising 13-items (C-M generation = 7 items, S-R change = 6 items). In study 3, a separate adult sample was recruited to firstly reconfirm (replicate) the two-factor structure within a new sample, and secondly to conduct criterion validity tests on the CMBQb. We examined criterion validity in relation to emotion regulation and emotion reactivity (e.g., [21]). If the CMBQb assesses C-M generation and S-R change beliefs in the hypothesised manner, scores reflecting greater C-M generation and lower S-R change beliefs should be related to more adaptive emotion regulation (higher cognitive reappraisal). Further, scores reflecting greater C-M generation beliefs and lower S-R change beliefs were hypothesised to be related to lower emotion reactivity. Finally, we determined floor (lowest possible score) and ceiling (highest possible score) effects as an additional marker of quality. As in the previous studies of the current paper, no S-R generation and C-M change (CMBQa) items were used.

### Participants

405 participants completed the CMBQb, and from this sample, a subsample of *n* = 149 participants completed additional questionnaires in order to complete criterion validity tests. Although we used an a priori participant:item ratio of 10:1 (e.g., [31]) we were more concerned with attrition in study 3 than in study 2 due to additional questionnaires, and thus recruited a greater number of participants. Prolific was used to recruit an adult sample following the same criteria and procedures as was used for the EFA (study 1) and CFA (study 2) studies. The blocklist facility in Prolific ensured that participants who had participated in the EFA and CFA studies could not take part in this study. A total of two respondents’ data were excluded due to poor data quality (i.e., straight line responding, unrealistic completion time). The final sample for CFA comprised 403 respondents (*M*age = 31.92; *SD*age = 8.50; female = 184; male = 219; Asian = 18; Black = 15; Mixed race = 6; White = 360; Other = 4). Ethical approval was granted from a University ethics committee, and informed written consent was gained from all participants.

### Procedure

All 403 participants completed the 13-item CMBQb, and the *n* = 149 subsample (*M*age = 30.95; *SD*age = 8.50; female = 70; male = 79) additionally completed the Emotion Regulation Questionnaire (ERQ; [46]), the Emotion Reactivity Scale (ERS; [47]), and the Affective Reactivity Index (ARI; [48]). The ERQ (cognitive reappraisal) indicated emotion regulation, and the ERS and ARI indicated emotion reactivity. Affective and emotion reactivity refers to the extent to which an individual experiences emotion in response to a wide array of stimuli (emotion sensitivity), intensely (emotion intensity), and for a prolonged time period (emotion persistence) [47]. Full measurement details can be found in Turner et al. (2021), but in the current study the CMBQb (C-M generation; M = 3.75, SD = .50, S-R change; M = 2.72, SD = .72) demonstrated Cronbach’s *α* of .79 for C-M generation, and .80 for S-R change. The ERQ cognitive reappraisal scale demonstrated a Cronbach’s *α* of .89, the ERS demonstrated Cronbach’s *α* of .87 for emotion sensitivity, .90 emotion intensity, and .90 emotion persistence, and the ARI demonstrated a Cronbach’s *α* of .83.

### Data analysis

Data were screened for outliers (standardized z values > 3.29), and outliers were Winsorized (*n* = 32 from 10,260 cases = .31%). Data were Winsorized for CMBQ cases (*n* = 20), and ARI cases (*n* = 12). First, the 13-items of the CMBQb (C-M generation = 7 items, S-R change = 6 items) were subjected to CFA using SEM in AMOS version 25, in which a correlated two-factor model was tested (Table 2). The same goodness of fit indices posited by Schermelleh-Engel et al. [49] were used to determine acceptable fit, and the same MI guidelines by Rossier et al. [50] were used. Then, following similar research (e.g., [39]), criterion validity was tested by examining Pearson’s bivariate correlation co-efficients (using SPSS version 25) between the CMBQb subscale scores, and ERQ (cognitive reappraisal) scores, and ARI, and ERS scores (Table 3). It was hypothesised that greater C-M generation and lesser S-R change scores would be significantly related (*p* < .05) to greater ERQ (cognitive reappraisal), and lower ARI and ERS scores.

**Table 2:** Item properties, internal consistency, inter-item correlations, and descriptives, of the 13-item CMBQb in study 3.

						Inter-item correlation	
Item		*β*	*R* ^2^	*α*	M(SD)	Range	M(SD)
	**C-M generation**			.79			
2	My thoughts about the situation cause me to feel these unpleasant emotions.	.53	.28		3.69(.77)	.264-.377	.323(.051)
3	How I feel is dictated by my thoughts about the situation.	.54	.29		3.88(.74)	.275-.377	.325(.035)
4	My emotions are caused by my thoughts about things around me.	.59	.35		3.68(.77)	.264-.396	.341(.054)
5	My thoughts about what happens to me makes me feel these unpleasant emotions.	.64	.40		3.66(.79)	.275-.462	.365(.062)
1	How I feel is dictated by my thoughts towards things that happen in my life.	.60	.37		3.69(.80)	.270-.482	.346(.081)
6	My emotions are caused by my thoughts about things that happen to me.	.66	.44		3.71(.76)	.272-.482	.372(.088)
7	My thoughts about things around me makes me feel how I feel.	.53	.28		3.90(.70)	.272-.376	.322(.044)
	**S-R change**			.80			
17	Only removing myself from the situation can alter how I feel.	.67	.45		2.79(1.09)	.171–687	.437(.190)
20	I can only change how I feel, by removing myself from the situation.	.65	.42		2.69(1.04)	.197–687	.433(.183)
16	In order to change how I feel, peoples’ actions towards me need to change.	.28	.08		2.63(.91)	.171-.463	.250(.120)
22	I can change how I feel, only by changing the situation I am in.	.59	.35		3.02(1.11)	.214-.518	.373(.108)
18	Only by changing how people act around me can I really change how I feel.	.61	.37		2.37(.95)	.382-.463	.437(.190)
19	Only by changing the situation, can I change how I feel.	.78	.61		2.84(1.02)	.203-.528	.445(.138)

*Note*. Item 16 (S-R change) had a low factor loading compared to other items, so should be investigated further in a different sample to indicate whether they are surplus to requirements or not.

**Table 3:** Study 3 Pearson’s correlation coefficients.

	M(SD)	1	2	3	4	5	6	7	8
1. C-M generation	3.75(.47)	-							
2. S-R change	2.74(.71)	-.10	-						
3. ERQ cognitive reappraisal	28.22(6.61)	.39**	-.19*	-					
4. PMH	26.78(5.38)	-.05	-.11	.34**	-				
5. ARI	2.98(2.72)	.13	.36**	-.22*	-.36**	-			
6. ERS sensitivity	15.02(5.64)	.12	.35**	-.13	-.47**	.66**	-		
7. ERS intensity	18.43(6.40)	.11	.29**	-.07	-.30**	.59**	.84**	-	
8. ERS persistence	20.08(7.28)	.12	.38**	-.19*	-.44**	.65**	.90**	.85**	-
9. ERS total	53.53(18.41)	.13	.36**	-.14	-.42**	.66**	.95**	.94**	.96**

*Note*. **p < .001,

*p < .01

## Results

### CFA

The 13-item two-factor model was a good fit, *χ*^2^ = 113.501, df = 61, *p* < .001, RMSEA = .046 (90% CI = .033–.059), CFI = .96, NFI = .92, TLI = .95, SRMR = .050. See Table 2 for factor loadings.

### Criterion validity

#### Emotion regulation

In the Pearson’s correlation analysis (Table 3), C-M generation scores were positively associated with cognitive reappraisal (*r* = .39, *p* < .001), and S-R change scores were negatively associated with cognitive reappraisal (*r* = -.19, *p* < .01).

#### Emotion reactivity

In the Pearson’s correlation analysis (Table 3) C-M generation scores were not related to ARI or ERS scores. Higher S-R change scores were related to greater emotion sensitivity (*r* = .35, *p* < .001, intensity (*r* = .29, *p* < .001), and persistence (*r* = .38, *p* < .001), and affective reactivity (*r* = .36, *p* < .001).

### Construct validity

#### Floor and ceiling effects

No more than 15% of a sample should obtain the top or bottom score [51]. In the current sample, for C-M generation one participant (0.2%) scored the lowest (1), and six participants (1.5%) scored the highest (5) possible score, and for S-R change six participants (1.5%) scored the lowest (1), and no participants scored the highest (5) possible score.

## Discussion

The results of study 3 confirmed the correlated two-factor structure of the 13-item CMBQb. Also, greater C-M generation scores and lesser S-R change scores were positively related to cognitive reappraisal, and greater S-R change scores were negatively related to affective and emotion reactivity, demonstrating criterion validity. However, C-M generation scores were unrelated to affective and emotion reactivity. Finally, descriptive statistics revealed acceptable floor and ceiling effects, which indicates some construct validity.

## Study 4

The focus of the current paper thus far has been on the validation of the CMBQb as a complimentary assessment of emotion beliefs to the already validated CMBQa [21]. Whilst the CMBQa assesses S-R generation and C-M change, the proposed CMBQb assesses SR-change and C-M generation. Given the overlapping theoretical ideas between the CMBQa and the CMBQb, there are some important questions remaining as to a) whether and to what extent the two factors with each of the CMBQ questionnaires can form a four-factor solution, b) whether and what extent these four factors can tell us anything about emotion regulation tendencies and emotion reactivity outcomes, and c) whether and to what extent the four factors can collapse into two composite subscales of S-R beliefs ([S-R generation + S-R change] / 2) and C-M beliefs ([C-M generation + C-M change] / 2). To phrase point c) above differently, does the distinction between generation and change matter to emotion regulation and reactivity outcomes, or is it more useful to consider a single S-R subscale and a single C-M subscale? As an extension to the idea of synthesising the two CMBQs down to two subscales, and as a fourth consideration, d) it is also possible to consider a single discrepancy value (C-M minus S-R, e.g., hedonic balance; [52]). It could be the extent to which C-M beliefs predominate over S-R beliefs, or vice versa, that is most important for emotion regulation and reactivity.

To address the four points above, in Study 4 CFA is conducted with the four-factors offered within the CMBQa and CMBQb, namely S-R generation and C-M change (CMBQa), and C-M generation and S-R change (CMBQb). Following CFA, criterion validity tests are conducted to examine the associations between the CMBQ factors (four separate factors, and two combined factors) and prominent self-regulation tendencies, and emotion reactivity outcomes. Scores reflecting greater C-M beliefs (generation and change separately, and combined) and lower S-R beliefs (generation and change separately, and combined) were hypothesised to be related to more adaptive emotion regulation tendencies and lower emotion reactivity. Furthermore, a CM-SR discrepancy score (or a CM-SR index), is calculated such that higher scores indicate greater relative C-M beliefs and lower relative S-R beliefs. We then correlate this CM-SR index with prominent self-regulation tendencies, and emotion reactivity outcomes. We hypothesised that a greater index score would be related to more adaptive emotion regulation tendencies and lower emotion reactivity. Finally, to reflect the potential conceptual similarity between change beliefs (S-R change, C-M change), and controllability beliefs offered in past research (e.g., [27]), we hypothesised that S-R and C-M change beliefs would be related to more adaptive emotion regulation tendencies and lower emotion reactivity.

### Participants

In a separate sample to the previous studies, *n* = 254 participants (*M*age = 32.49; *SD*age = 8.47; female = 114; Asian = 13; Black = 12; Mixed race = 4; White = 225) took part in the current study. Prolific was used to recruit an adult sample following the same criteria and procedures as was used for all previous studies in the current paper. Ethical approval was granted from a University ethics committee, and informed written consent was gained from all participants.

### Measures

As in study 3, participants completed the CMBQb (13-item; C-M generation = 7 items, S-R change = 6 items), the ARI (affective reactivity; Cronbach’s *α* = .82), and the ERS (emotion reactivity; Cronbach’s *α* of .84 for emotion sensitivity, .88 emotion intensity, and .88 emotion persistence). Participants also completed the following questionnaires.

#### Emotion beliefs

The CMBQa [21] comprises 15-items and assesses S-R generation beliefs (8-items) and C-M change beliefs (7-items). The CMBQa demonstrates good construct, criterion, and test-retest validity [21].

#### Emotion regulation tendencies

The regulation of emotion systems survey (RESS; [53]) was used to measure reappraisal (4-items; Cronbach’s *α* = .88; e.g., “Thinking of other ways to interpret the situation”), suppression (4-items; Cronbach’s *α* = .83), distraction (4-items; Cronbach’s *α* = .92; e.g., “Doing something else to distract myself”), and engagement tendencies (4-items; Cronbach’s *α* = .83; e.g., “Vocalizing how I was feeling”). In addition to the RESS, we used acceptance (4-items; Cronbach’s *α* = .77; e.g., “I think that I have to accept the situation”), and putting into perspective (4-items; Cronbach’s *α* = .88; e.g., “I think that it could have all been much worse”) questions from the cognitive emotion regulation questionnaires (CERQ; [54]). We wanted to limit participant burden, and therefore we were selective over the variables we measured from the RESS and CERQ, choosing the variables that were most theoretically in-line with S-R and C-M beliefs.

### Data analysis

Data were screened for outliers (standardized z values > 3.29), and outliers were Winsorized (*n* = 21 from 22,352 cases = .09%). Data were Winsorized for CMBQa and CMBQb cases (*n* = 15), ARI cases (*n* = 5), and acceptance case (n = 1). First, the 13-items of the CMBQb (C-M generation = 7 items, S-R change = 6 items) and the 15 items of the CMBQa (S-R generation = 8 items, C-M change = 7 items) were subjected to CFA using SEM in AMOS version 25, in which a correlated four-factor model (28 items) was tested. The same goodness of fit indices posited by Schermelleh-Engel et al. [49] were used to determine acceptable fit, and the same MI guidelines by Rossier et al. [50] were used. Then, criterion validity was tested by examining Pearson’s bivariate correlation co-efficients (using SPSS version 25) between the CMBQa and CMBQb subscale scores, and RESS (reappraisal, suppression, distraction, engagement) scores, CERQ (acceptance and putting into perspective) scores, and ARI, and ERS scores. It was hypothesised that greater C-M scores and less S-R scores, and greater CM-SR index scores, would be significantly related (*p* < .05) to greater reappraisal, engagement, acceptance, and putting into perspective (adaptive emotion regulation), and lower suppression and distraction (maladaptive emotion regulation). It was also hypothesised that greater C-M scores and less S-R scores, and greater CM-SR index scores, would be significantly related (*p* < .05) to less affective (ARI) and emotion (ERS) reactivity scores.

## Results

### CFA

The 28-item two-factor model was a somewhat acceptable fit, *χ*^2^ = 590.954, df = 339, *p* < .001, RMSEA = .054 (90% CI = .047–.061), CFI = .91, NFI = .81, TLI = .90, SRMR = .064. See Table 4 for factor loadings. Similar to study 2 findings, item 16 of S-R change demonstrated a particularly low factor loading (.32), but all other loadings were between .48 and .79.

**Table 4:** Item properties, internal consistency, inter-item correlations, and descriptives, of the 28-item CMBQc (CMBQa and CMBQb combined) in study 4.

						Inter-item correlation	
Item		*β*	*R* ^2^	*α*	M(SD)	Range	M(SD)
	**S-R generation**			.88			
	My feelings are entirely determined by peoples’ actions towards me.	.68	.46		2.30(.93)	.277-.584	.467(.101)
	What happens to me entirely dictates how I feel.	.79	.62		2.97(1.07)	.385-.685	.532(.112)
	My emotions are caused entirely by the things that happen to me.	.77	.59		2.80(1.07)	.383-.744	.530(.133)
	My emotions are completely dictated by what happens to me.	.75	.56		2.80(1.00)	.380-.744	.523(.138)
	My emotions are caused entirely by others’ actions towards me.	.60	.36		2.33(.99)	.311-.614	.445(.101)
	My emotions are entirely caused by what people do around me.	.67	.45		2.37(1.00)	.343-.614	.489(.089)
	My feelings are completely controlled by the situation I am in.	.67	.45		2.65(1.01)	.355-.504	.449(.064)
	How I feel is completely dictated by the things that happen to me in my life.	.49	.24		3.09(1.06)	.277-.444	.357(.054)
	**C-M change**			.81			
	To control my emotions, I need to change the way I think.	.48	.23		3.81(.85)	.266-.399	.310(.057)
	To change how I feel, I need to change what I think about things around me.	.56	.31		3.72 (.93)	.232-.399	.345(.063)
	To change how I feel, I can change my thoughts about the situation.	.66	.43		3.66(.83)	.355-.486	.402(.051)
	Because I can choose to think differently, I can choose to feel differently about the situation.	.63	.39		3.48(1.00)	.232-.528	.383(.121)
	To change how I feel, my thoughts about the situation need to change.	.58	.34		3.65(.87)	.275-.427	.365(.058)
	I can change my emotions by changing how I think about the situation.	.74	.55		3.76(.73)	.311-.537	.440(.087)
	Thinking differently about the situation will change how I feel.	.68	.46		3.78(.86)	.252-.528	.403(.116)
	**C-M generation**			.80			
2	My thoughts about the situation cause me to feel these unpleasant emotions.	.57	.32		3.69(.76)	.257-.433	.347(.070)
3	How I feel is dictated by my thoughts about the situation.	.60	.36		3.84(.79)	.333-.433	.372(.042)
4	My emotions are caused by my thoughts about things around me.	.57	.33		3.69(.79)	.257-.416	.348(.056)
5	My thoughts about what happens to me makes me feel these unpleasant emotions.	.66	.44		3.65(.81)	.339-.498	.397(.063)
1	How I feel is dictated by my thoughts towards things that happen in my life.	.61	.37		3.72(.79)	.313-.461	.360(.058)
6	My emotions are caused by my thoughts about things that happen to me.	.68	.46		3.72(.75)	.302-.498	.396(.077)
7	My thoughts about things around me makes me feel how I feel.	.55	.31		3.90(.68)	.302-.417	.349(.051)
	**S-R change**			.80			
17	Only removing myself from the situation can alter how I feel.	.63	.40		2.75(1.13)	.152-.748	.451(.196)
20	I can only change how I feel, by removing myself from the situation.	.60	.36		2.72(1.04)	.139-.748	.434(.202)
16	In order to change how I feel, peoples’ actions towards me need to change.	.32	.10		2.63(.91)	.139-.441	.213(.115)
22	I can change how I feel, only by changing the situation I am in.	.64	.41		2.99(1.10)	.174-.534	.370(.117)
18	Only by changing how people act around me can I really change how I feel.	.68	.46		2.35(.94)	.411-.441	.424(.011)
19	Only by changing the situation, can I change how I feel.	.73	.54		2.83(1.06)	.157-.544	.437(.147)

*Note*. Item 16 (S-R change) had a low factor loading compared to other items, so should be investigated further in a different sample to indicate whether they are surplus to requirements or not.

### Criterion validity

#### Emotion regulation

In the Pearson’s correlation analysis (Table 5), S-R generation and S-R change scores were negatively associated with reappraisal (respectively *r* = -.15 and *r* = -.20, *p* < .01), and positively associated with suppression (respectively *r* = .24 and *r* = .28, *p* < .001). S-R change was also positively associated with distraction (*r* = .13, *p* < .05). C-M generation scores were positively associated with engagement (*r* = 16, *p* < .02), acceptance (*r* = .22, *p* = .001), and putting into perspective (*r* = .27, *p* < .05) and C-M change scores were positively associated with reappraisal (*r* = .20, *p* < .01), acceptance (*r* = .25, *p* < .001), and putting into perspective (*r* = .27, *p* < .001), and negatively associated with suppression (*r* = -.16, *p* < .01).

**Table 5:** Study 4 Pearson’s correlation coefficients (C-M comp = composite C-M score, S-R comp = composite S-R score, perspective = putting into perspective).

	M(SD)	1	2	3	4	5	6	7	8	9	10	11	12	13	14	15	16
1. C-M generation	3.75(.52)	-															
2. C-M change	3.70(.59)	.49**	-														
3. S-R generation	2.67(.75)	.03	-.21**	-													
4. S-R change	2.71(.73)	-.02	-.18*	.68**	-												
5. C-M comp	3.72(.47)	.84**	.88**	-11	-.11	-											
6. S-R comp	2.69(.68)	.01	-.21**	.92**	.91**	-.12	-										
7. CM-SR index	1.03(.86)	.45**	.64**	-.78**	-.77**	.64**	-.84**	-									
8. Reappraisal	3.03(.93)	.06	.20*	-.15*	-.20*	.16*	-.19**	.23**	-								
9. Suppression	2.67(.87)	-.06	-.16*	.24**	.28**	-.13*	.29**	-.30**	-.04	-							
10. Distraction	3.07(.99)	.09	.00	.04	.13^†^	.05	.09	-.05	.16*	.33**	-						
11. Engagement	2.75(.88)	.16*	.07	.05	-.03	.13*	.01	.06	.18*	-.37**	.04	-					
12. Acceptance	3.32(.69)	.22*	.25**	-.10	-.11	.27**	-.11	.23**	.26**	.16*	.12	-.09	-				
13. Perspective	3.31(.98)	.14^†^	.27**	-.06	.05	.25**	-.01	.14*	.30**	.01	.16^†^	-.02	.37**	-			
14. ARI	2.80(2.62)	.03	-.13^†^	.31**	.25**	-.06	.30**	-.27**	-.13^†^	.24**	.17*	.21**	-.08	-.01	-		
15. ERS sensitivity	14.35(5.19)	.12	-.06	.31**	.28**	.03	.32**	-.24**	-.10	.15^†^	.11	.38**	-.13^†^	-.07	.62**	-	
16. ERS intensity	18.05(6.05)	.15^†^	-.02	.18*	.12	.06	.17**	-.10	.00	.07	.15^†^	.49**	-.08	-.12	.52**	.81**	-
17. ERS persistence	19.81(7.13)	.11	-.04	.33**	.31**	.03	.35**	-.25**	-.12	.16*	.13^†^	.35**	-.11	-.11	.62**	.88**	.80**

*Note*. **p < .001,

*p < .01,

^†^*p* < .05. ARI = affective reactivity index; ERS = emotion reactivity scale.

For S-R composite and C-M composite scores, Pearson’s correlation analysis revealed that S-R composite scores were negatively associated with reappraisal (*r* = -.19, *p* < .001) and positively associated with suppression (*r* = .29, *p* < .001). C-M composite scores were positively associated with reappraisal (*r* = .16, *p* < .01), engagement (*r* = .13, *p* < .05), putting into perspective (*r* = .25, *p* < .001), and acceptance (*r* = .27, *p* < .001), and negatively associated with suppression (*r* = -.13, *p* < .05). In addition, Pearson’s correlation analysis revealed that CM-SR index scores were positively associated with reappraisal (*r* = .23, *p* < .001), putting into perspective (*r* = .14, *p* < .005), and acceptance (*r* = .23, *p* < .001), and negatively associated with suppression (*r* = -.30, *p* < .001).

#### Emotion reactivity

In the Pearson’s correlation analysis (Table 5), S-R generation and S-R change scores were positively associated with affective reactivity (respectively *r* = .31, and *r* = .25, *p* < .001) and emotion persistence and sensitivity (respectively *r* = .33 and r = .31, *p* < .001). S-R generation was positively associated with emotion intensity (*r* = .18, *p* < 01). C-M generation scores were positively associated with emotion intensity (*r* = .15, *p* < 02), and C-M change scores were negatively associated with affective reactivity (*r* = -.13, *p* < .05).

For S-R composite and C-M composite scores, Pearson’s correlation analysis revealed that S-R composite scores were positively associated with affective reactivity (*r* = .30, *p* < .001) and emotion persistence (*r* = .35, *p* < .0010 and sensitivity (*r* = .32, *p* < .001), and emotion intensity (*r* = .17, *p* = .008). C-M composite scores were not related to ARI or ERS scores. In addition, Pearson’s correlation analysis revealed that CM-SR index scores were negatively related to affective reactivity (*r* = -.27, *p* < .001) and emotion persistence (*r* = -.25, *p* < .001) and sensitivity (*r* = -.24, *p* < .001).

## Discussion

In study 4 the four-factor structure of the combined CMBQ (CMBQc) was tested for factorial and criterion validity. CFA results indicated a somewhat acceptable fit to the data, with one item (item 16) poorly loading onto the S-R change factor. It was also found that scores indicating greater C-M generation and change beliefs, and lower S-R generation and change beliefs, were related to more adaptive, and less maladaptive, emotion regulation tendencies. In addition, there was some evidence that greater C-M change beliefs, and lower S-R generation and change belief, were related to better affective and emotion reactivity outcomes. However, contrary to expectations, greater C-M generation beliefs were related to greater emotion intensity. Whilst this association was weak (*r* = .13), it is still worthy of note, and might reflect that C-M generation beliefs are not sufficient alone and may require C-M change beliefs to have an impact upon emotional outcomes. It is also possible that believing that emotions are generated by one’s own thoughts may lead to rumination that exacerbates emotion intensity, or encourages self-blame for one’s emotions and thus could be generative of further negative emotion. Of course, these explanations are speculative at this point, and more date are required to be able to test these conjectures.

Study 4 also moved the research into the CMBQc forward in terms of emotion regulation tendency assessment. Whereas Turner et al. [21] and studies 1–3 focussed on reappraisal due to its clear theoretical convergence with S-R and C-M beliefs, in study we also measured acceptance, engagement, distraction, suppression, and putting into perspective.

In study 4 we also formulate an S-R composite score and a C-M composite score, and a CM-SR index score (C-M minus S-R) to examine the utility of synthesising down the four factors of the CMBQc. Broadly, greater S-R and lower C-M composite scores, and greater CM-SR index scores (indicting higher C-M beliefs relative to S-R beliefs) were associated with more adaptive, and less maladaptive, emotion regulation tendencies, and better affective and emotion reactivity outcomes. Although, there are some nuances in these results that need to be elaborated. First, the associations between the S-R and C-M composite scales and affective and emotion reactivity indicate that it is S-R beliefs that are related to greater reactivity, rather than C-M beliefs being related to lower reactivity. This is consistent with the C-M generation and C-M change scores, which also do not relate, or are weakly related, to reactivity scores, whilst S-R generation and S-R change scores reflect stronger associations with reactivity scores.

In contrast, for the results concerning emotion regulation tendencies, C-M composite scores were associated with a variety of strategies including greater reappraisal, engagement, acceptance, and putting things into perspective, and lower suppression, whilst S-R composite scores were only related to lower reappraisal and higher suppression. It could be that S-R beliefs are not conducive to, or are prohibitive of, the particular emotion regulation strategies we measured. The CM-SR index scores support the findings for the separate S-R and C-M composite scores, in that a greater CM-SR index score was not related to distraction or engagement, but was related to the other emotion regulation strategies. As such, it appears that whilst there are some differences in the findings across the four separate S-R/C-M generation and change scores, S-R and C-M composite, and CM-SR index scores, there is a largely consistent narrative that when the results are taken together. It appears that the emotion beliefs combination that is most associated with adaptive emotion regulation tendencies and affective and emotion reactivity is one of high C-M beliefs and low S-R beliefs. Whilst nuanced relationships between CMBQ subscales and emotion regulation and reactivity are apparent, there appears to be some justification for utilising a CM-SR index. As such, it could be argued that it is the extent to which C-M beliefs predominate over and above S-R beliefs that are indicative of greater adaptive emotion regulation tendencies and lower affective and emotion reactivity.

Results of this more expansive emotion regulation assessment reveal some useful findings that are in line with what we would expect given the proposed implications of S-R and C-M beliefs. For example, it makes sense that S-R is related to less reappraisal, and more related to suppression and distraction. If I believe that emotions are only situationally determined, then a viable recourse for emotion regulation might be to distract myself from the situation and or suppress my emotion, since I might not see the viability of cognitive reappraisal. In contrast, it makes sense that C-M is related to more reappraisal, engagement, acceptance and putting into perspective, and less related to suppression. If I believe that emotions are cognitively mediated (C-M), then my recourse for emotion regulation might viably be cognitive reappraisal which allows me to engage with my emotions, rather than suppress them. I am also more likely to attempt to put the situation into perspective, since this strategy relies on reappraisal. Also, if I have high C-M beliefs and low S-R beliefs (which is not always necessarily the case) my focus might be less on changing the situation and more on accepting it, because I believe in that cognitive change leads to emotion change, rather than situation change, so focussing on the situation presents less emotion regulation potential. Of course, it is possible to endorse both C-M and S-R beliefs, and it is not necessarily the case that just because I have high C-M beliefs that I will have low S-R beliefs, and indeed, data in the present study reveals only a small correlation between C-M and S-R scores. In sum, the results of study 4 offers some support for the CMBQc, and extend the research by introducing C-M and S-R composite scores, and the CM-SR index.

## General discussion

The chief purpose of the present paper was to develop and undertake initial validity testing of two emotion beliefs, namely C-M generation beliefs and S-R change beliefs. In studies 1–3 of the current paper, the factor structure of the CMBQb emerged and was validated through EFA (study 1) and CFA (study 2), confirmed in an additional CFA (study 3), and criterion validity testing (study 3). Greater C-M generation beliefs and lower S-R change beliefs were related to more adaptive emotion regulation tendencies. Greater S-R change beliefs, but not C-M generation beliefs, were related to greater emotional and affective reactivity. Results for studies 1–3, particularly for S-R change beliefs, are noteworthy when considering the proposed shortcomings of an S-R viewpoint of emotion (e.g., [13]). In other words, the finding that S-R change beliefs are related to less reappraisal and poorer emotional outcomes lends support to the idea that a reliance on situational change (or modification), and or the belief that emotions are the result of external events only, might be deleterious to emotion regulation and emotion reactivity.

A secondary purpose of the current paper was to test the validity of the CMBQc, which is a combination of CMBQa [21], and CMBQb (emergent in the studies 1–3 of the present paper). Turner et al. [21] found support for the two emotion beliefs of S-R generation and C-M change (CMBQa), but not for C-M generation and S-R change. That is, EFA and CFA in Turner et al. (2021) revealed two factors comprising S-R generation and C-M change items, which excluded all C-M generation and S-R change items. Therefore, in studies 1–3 of the present paper we took the excluded C-M generation and S-R change items from Turner et al. (2021) and applied separate validity tests to examine the factor structure and criterion validity of the CMBQb. Then in study 4 of the present paper we combined CMBQa and CMBQb to test the proposed four-factor structure of a CMBQc. The 28-item CMBQc offers an assessment of all four superordinate emotion beliefs proposed by Turner et al. [21]. That is, CMBQc assesses C-M generation beliefs (emotions are cognitively mediated), C-M change beliefs (changes in cognition lead to emotion change), S-R generation beliefs (emotions are caused by events), and S-R change beliefs (changes in the situation lead to emotion change).

The results of the present paper when considered alongside Turner et al. [21] provide a more comprehensive representation of how S-R and C-M beliefs might influence emotion regulation and emotion reactivity. Specifically, S-R beliefs (generation and change) were related to less adaptive emotion regulation tendencies, and greater emotion reactivity. In contrast, C-M generation and change beliefs were related to more adaptive emotion regulation tendencies, and C-M change beliefs were related to lower emotion reactivity. However, it remains unclear as to whether C-M generation beliefs are beneficial for emotion reactivity. In study 3, C-M generation beliefs were unrelated to affective and emotion reactivity, and in study 4, greater C-M generation beliefs were related to greater emotion intensity, which was counter to expectations. Therefore, whilst the current paper offers some initial promising findings for the validity of the CMBQc, more research is needed in order to establish exactly how the four superordinate emotion beliefs relate to and interact with emotion regulation to predict affective and emotion reactivity.

S-R generation and change beliefs reflect the notion that emotion is dependent upon situational events, whilst C-M generation and change beliefs reflect the notion that emotion is dependent upon the one’s *cognitions about* situational events. There is some clear convergence between the ideas of S-R and C-M beliefs, and the Adversity-Consequences and Beliefs-Consequences thinking present in REBT, and other CBTs. In REBT, clients are encouraged to understand that their emotions are cognitively mediated (Beliefs-Consequences), rather than the sole result of external events (or stimuli; Adversity-Consequences thinking). But the S-R and C-M emotion beliefs captured in the CMBQc also contain two additional and related ideas; (a) emotion is generated by the situation (S-R generation), or by one’s cognitions about the situation (C-M generation), and (b) emotion is regulated by changing the situation (S-R change), or by changing one’s cognitions (C-M change). Thus, one aspect involves a set of processes related to the *generation* of emotion, and a second aspect involves a different set of processes related to the *management* of emotion (e.g., [28, 29]). However, what remains to be examined concerning the CMBQc, is the extent to which the four factors interact with *one another* to influence emotion regulation and reactivity.

Some have argued that emotion generation and regulation may be inseparable [18], but distinguishing between emotion reactivity from emotion regulation is useful for analysing processes, individual differences, and developing interventions [30], but whilst the separation is practically appealing, it must be considered that emotion and emotion regulation cannot be separated because they are tightly intertwined [28]. As such, in the current study, and in Turner et al. [21], generation and change beliefs are separate emotion beliefs. If one holds C-M generation beliefs then it is perhaps more likely that one would hold C-M change beliefs, and consequently would be more likely to apply cognitive reappraisal. In contrast, if one holds S-R generation beliefs then it is perhaps more likely that one would hold S-R change beliefs, and consequently would be less likely to apply cognitive reappraisal. Indeed, cognitive change might be more effective in decreasing negative affect when emotions are generated in a top-down (reflecting C-M generation), rather than a bottom-up (reflecting S-R generation), fashion [55]. However, these hypotheses are yet to be tested, but now with the instantiation of questionnaires that assess the four proposed emotion beliefs, in future research it is possible to undertake such an investigation.

Indeed, it is of course possible that one’s generation beliefs are not indicative of change beliefs, but correlations between generation and change factors with S-R and C-M suggests orthogonality because S-R generation is positively related to S-R change, and C-M generation is positively related to C-M change. Also, C-M composite scores are unrelated to S-R composite scores, indicating some independence. However, C-M change is negatively related to both S-R generation and S-R change, indicating that rather than being independent, S-R beliefs are at odds but related with C-M change beliefs. The orthogonality of S-R and C-M beliefs needs to be studied further, but preliminary evidence is indicative of orthogonality. If this is the case, then it is not enough in practice to merely weaken a client’s S-R beliefs, one must also work to strengthen and reinforce C-M beliefs. One cannot assume that low S-R beliefs is illustrative of high C-M beliefs.

The composite C-M and S-R subscales appear to offer some value, in that they are related to emotion regulation and reactivity outcomes, but what is lost in the composite scores are the specific subscales for generation and change, which might differentially predict emotion regulation. Indeed, when comparing generation and change beliefs, it is perhaps the change beliefs that we would expect to be related to actual emotion regulation attempts. If I have strong C-M change beliefs then it stands to reason that I would be more likely to attempt cognitive change, whereas if I have strong S-R change beliefs then I might be more likely to attempt situation selection and or situation modification (e.g., [1]). Also, strong C-M change believers may be more likely to also believe that *they* can change their own emotions, compared to S-R change believers, who might be more likely to endorse environmental mechanisms in shaping their emotions (i.e., being around the right people). Indeed, in study 4 of the present paper, it is C-M change, not C-M generation, that related to emotion regulation tendencies, perhaps indicating for the C-M subscales, that it is C-M change that is the more likely to predict cognitive reappraisal attempts.

In the present study, greater S-R change beliefs related to greater suppression and lower cognitive reappraisal, but also worse emotion reactivity outcomes. Thus, an individual with high S-R change believes might be less likely to apply cognitive reappraisal. However, this does not mean that S-R change believers would be unable to regulate their emotions in situ via situation change. The limitations of a situation change approach are that one cannot always select or modify a situation, and avoidance of emotionally provocative situations, whilst potentially effective in the short-term, is no long-term strategy for emotional health.

In studies 3 and 4 of the current paper, it was quite clear that S-R change was related to less adaptive emotion regulation tendencies and poorer emotion reactivity tendencies, whilst C-M change in the current paper was related to more adaptive emotion regulation tendencies and greater affective reactivity, and in previous research has been related to poorer emotion reactivity. Thus, S-R change and C-M change do not appear to tell us the same thing, and as such, should be considered as distinct subscales. Similarly, S-R generation and C-M generation scores do not seem to relate to one another and do not relate to adaptive emotion regulation tendencies in the same way. Thus, there seems little rationale for conflating the two generation subscales. So whilst S-R and C-M composite scores, and the CM-SR index might be informative concerning emotion regulation and reactivity, and offer a concise way to perform analyses with the CMBQc, it is recommended that researchers utilise the four separate subscales for the purposes of predicting emotion regulation attempts and emotion reactivity.

The findings of the current paper, and the proposed of S-R and C-M emotion superordinate beliefs per se, should be considered alongside the foundational work that has already been conducted for other emotion beliefs. Specifically, an important line of future enquiry is to understand how S-R and C-M beliefs interacts with the goodness and controllability beliefs that have been the subject of past research (e.g., [22, 27]) and conceptualisation (Ford & Gross, 2018). Theoretically one could venture several hypotheses worth testing regarding S-R, C-M, goodness, and controllability beliefs. For example, we may expect those reporting greater S-R beliefs, alongside lower C-M, lower goodness (emotions are not good), and lower controllability (emotions are not controllable) beliefs, to report poorer psychosocial outcomes and worse emotionality (more emotional intensity, sensitivity, and persistence). Second, we may expect those reporting greater C-M change and S-R change beliefs to also report greater controllability beliefs, given that beliefs concerning change may be related to, dependent upon, or antecedent to, beliefs concerning controllability–I cannot change something that I cannot control.

However, from an emotion regulation perspective the picture may look a little more complex. It could be argued that greater C-M and controllability beliefs, alongside lower S-R beliefs, would lead to cognitive reappraisal attempts in order to regulate emotion. But goodness beliefs may determine whether emotion regulation is even attempted, because believing that an emotion is good may decrease the likelihood that the emotion is identified as needing regulation [17]. So, I may believe that the emotion is generated and regulated via C-M rather than S-R, and that emotions are controllable, but because I believe that the emotion is good, I may not attempt cognitive reappraisal because I do not desire to regulate the emotion. Thus, I may fail to regulate a controllable and dysfunctional emotion, because I view that emotion as good. Therefore, future research could focus on collected data across C-M, S-R, goodness, and controllability emotion beliefs and apply latent profile analysis (e.g., [56, 57]) to explore emotion beliefs profiles, and how these profiles relate to emotion regulation and reactivity outcomes.

In addition to goodness and controllability emotion beliefs, there may be other individual differences that are related to C-M and S-R beliefs. One potential avenue for further investigation would be to assess locus of control alongside C-M and S-R beliefs. Locus of control was posited by John Rotter [58, 59], and individuals can have an internal (we have the ability to control what happens to us) or external locus on control (what happens to us is determined by external forces). In relation to C-M and S-R beliefs it could be that those who endorse high internal locus of control will also endorse high C-M beliefs given that both ideas indicate that we ourselves can determine our destiny, emotional and otherwise. Indeed, one study [60] found that greater external locus of control was related to greater difficulties in regulating emotions and greater adaptive cognitive emotion regulation strategies.

The practical implications of the present study, alongside the findings of Turner et al. (2021), are manifold. In line with second-wave CBTs ([15, 61]), it is of course helpful to encourage clients to recognise the role their cognitions play in the aetiology and regulation of their emotions. C-M beliefs reflect greater volition of emotions, because cognitive change is often more possible when compared to stimulus or situation change (reflected by S-R beliefs). Indeed, given the weight of evidence in support of cognitive reappraisal, it is difficult to see the downsides of encouraging beliefs (like C-M beliefs) that make cognitive reappraisal more likely. It is not that clients are to blame for the external stimuli that often trigger emotion. Rather, we are suggesting that encouraging clients to understand that they can take some control of how their emotions occur and are regulated, will be beneficial for emotion reactivity and regulation. In line with second-wave CBT theory and practice, specifically rational emotive behaviour theory (REBT), we argue that by believing that emotion generation and change is cognitively mediated, one is more able to regulate one’s emotions. Thus, interventions informed by second wave CBTs should include a meaningful discussion with the client concerning their emotion beliefs, with a view to dissuading S-R beliefs and encouraging C-M beliefs. Alongside information psychoeducation concerning emotion beliefs, the CMBQ could be used as a marker of client A-C and B-C thinking pre-, post-, and during CBT to assess emotion belief change, and to assess whether these changes are related to therapeutic outcomes.

The above practical implications notwithstanding, just because an individual endorses C-M beliefs does not mean that they will execute cognitive reappraisal in everyday life, or in specific emotionally provocative situations. There are many factors to consider when predicting the selection and execution of emotion regulation strategies (e.g., social context; [62], regulation goals; [63]). For example, English et al. [64] found that reappraisal is used more when regulating for hedonic reasons (e.g., to feel better; pro-hedonic regulation), and were associate with particular instrumental goals such as getting work done. So, even if I have a strong emotion belief in C-M, my regulation goals will in part inform my selection of emotion regulation strategies. In other words, the influence of emotion beliefs upon emotion regulation has to be considered alongside the reasons why an individual is trying to change an emotion and also what they are trying to change (e.g., down or up regulation), which can be socially informed [19]. As such, C-M and S-R are just one potential piece of a complex puzzle that determined actual emotion regulation.

### Limitations of the present study

The studies concerning the CMBQc reflect cross-sectional validity, in that any associations between S-R and C-M beliefs and emotion regulation and reactivity are atemporal. Additional tests are required that assess the ability of the CMBQc to predict emotion regulation and reactivity in-situ, via experimental and longitudinal studies. It is not known to what extent C-M and S-R beliefs influence emotion outcomes overtime, or in reaction to acute emotive stimuli. This is important because data concerning the CMBQ thus far has relied upon cross-sectional data that has been collected from participants who are not undergoing an emotional episode. Therefore, there is little to indicate that S-R and C-M beliefs will have any impact on in-situ emotion regulation and reactivity.

Furthermore, the implications of C-M and S-R beliefs across different samples should be explored, such as in adolescent, athletic, student, military, and pathological samples. It is not clear whether an individual’s cultural setting, occupational context, or health, may influence holding of C-M and S-R beliefs, or to what extent it may determine the relationships between emotion beliefs and emotion regulation and reactivity. For example, if an individual’s occupational context dictates an S-R perspective on emotion then the individual may be very apt at regulating emotion via situation selection and or modification, rather than consciously employing cognitive reappraisal, and thus may not be as emotionally reactive as one might assume given their endorsement of S-R beliefs.

Further factor analyses should also be used with additional samples to confirm the four-factor structure of the CMBQc. Indeed, in study 4 the four-factor model yielded only somewhat acceptable fit indices, with one item showing a particularly low factor loading. Of course, to achieve better fit we could remove further items with lower relative factor loadings. However, we chose to be more conservative with item removal given the early stages of research into the CMBQ, and future research can explore if shortening the CMBQ yields greater model fit. In addition, a second order factor structure cannot be ruled out at this stage. That is, it might be that the CMBQc is best reflected in a S-R latent (S-R generation and S-R change) and C-M latent (C-M generation and C-M change) structure. It is important to undertake separate data collection to examine additional factor structures and not rely too heavily on data already collected, because the findings of cross-sectional research can be dependent upon the cohort in which the study was conducted. Therefore, further psychometric testing of the CMBQc is required. Lastly, the sample demographics of the studies in the current paper represent predominantly white United Kingdom citizens. This may be a function of the sampling methods we used (Prolific.ac), but future research should include a more diverse population to offer better representation across ethnicities.

## Conclusion

Across studies 1–3 the initial validity and reliability tests concerning the CMBQb reveal a correlated two-factor structure indicating C-M generation and S-R change beliefs. Results suggest that higher C-M generation lower S-R change beliefs relate to more adaptive emotion regulation, and that greater S-R change beliefs are related to poorer emotion reactivity. Study 4 brought the CMBQa and CMBQb together, revealing a correlated four-factor structure, and some criterion validity. The CMBQc is in need of further examination to confirm and or refine the factor-structure, and ascertain its predictive validity.

## Supporting information

S1 File Exploratory Factor Analysis (EFA) procedures [21, 32–34, 65–68]. (DOCX)

S2 File First EFA iteration including all 24 item numbers and text, factor loadings (bold text in boxes), and cross-loadings. (DOCX)

S3 File Confirmatory Factor Analysis (CFA) and test of bifactor models [21, 44, 45, 49, 50, 67, 69–76]. (DOCX)

S4 File Model fit indices for the correlated and bifactor, two-factor models. (DOCX)

S5 File Correlated and bifactor two-factor item loadings, Relative Parameter Bias (RPB), and Item Explained Common Variances (IECV). (DOCX)

S1 Data (SAV)

S2 Data (SAV)

S3 Data (SAV)

S4 Data (SAV)

S5 Data (SAV)
