# Supplementary material for: Knowing your ABCs: Extending the assessment of stimulus-response (S-R) and cognitive-mediation (C-M) beliefs
Source: PLoS One. 2022 Jun 14;17(6):e0269928. doi: 10.1371/journal.pone.0269928 (PMC9199960; doi:10.1371/journal.pone.0269928)
Supplement: S1 File — (DOCX) [file pone.0269928.s001.docx]

Supplementary file 1: Exploratory Factor Analysis (EFA) procedures

The procedure followed Turner et al. [21], and based on theory that informed item generation, we expected C-M generation and S-R change beliefs to be negatively correlated, so we used oblique rotation using Direct Oblimin with Kaiser Normalization. We used parallel analysis, the Kaiser criterion, visual inspection of the Scree plot, and the proportion of variance, alongside theoretical considerations to inform factor retention [32]. It is important to apply qualitative judgement to evaluate factor appropriateness in relation to the intended factor structure (e.g., [65]) and to be guided by theory to provide the results that make the most sense [66]. We wanted to pragmatically arrive at a final set of items that clearly assess theoretically derived emotion beliefs, so we were stringent on item retention so that the questionnaire could be reduced to a reasonable item-number. Therefore, in determining which items to retain, we applied the .6/.3 rule whereby an item was considered for retainment if it had a primary loading above .60, secondary loading (cross-loading) of below .30 [67], and a communality of over .40 [34]. In addition, we inspected inter-item correlations that exceeded .60 and for the correlated pair of items we removed the item that had greater inter-item correlations with other items. Factor analysis and associated item deletion was conducted iteratively, removing items one at a time, and repeating the EFA [33], [68].
