# Supplementary material for: Knowing your ABCs: Extending the assessment of stimulus-response (S-R) and cognitive-mediation (C-M) beliefs
Source: PLoS One. 2022 Jun 14;17(6):e0269928. doi: 10.1371/journal.pone.0269928 (PMC9199960; doi:10.1371/journal.pone.0269928)
Supplement: S2 File — (DOCX) [file pone.0269928.s002.docx]

Supplementary file 2. First EFA iteration including all 24 item numbers and text, factor loadings (bold text in boxes), and cross-loadings.

|  |  | Factors |  | Eigan values | % variance explained |
| --- | --- | --- | --- | --- | --- |
| Item number | Item text | 1 | 2 |  |  |
| 1 | My emotions are caused by my thoughts about things around me. | **0.683** | -0.014 | 6.20 | 25.69 |
| 2 | How I feel is dictated by my thoughts about the situation. | **0.659** | -0.035 |  |  |
| 3 | My thoughts about what happens to me makes me feel these unpleasant emotions. | **0.658** | -0.030 |  |  |
| 4 | My thoughts about the situation cause me to feel these unpleasant emotions. | **0.653** | -0.017 |  |  |
| 5 | How I feel is dictated by my thoughts towards things that happen in my life. | **0.644** | -0.051 |  |  |
| 6 | My emotions are caused by my thoughts about things that happen to me. | **0.624** | -0.034 |  |  |
| 7 | My thoughts about things around me makes me feel how I feel. | **0.597** | -0.001 |  |  |
| 8 | My emotions are caused by the way I think about things that happen in my life. | **0.574** | -0.162 |  |  |
| 9 | It is my thoughts about the situation, rather than the situation alone, that causes my emotions. | **0.536** | -0.150 |  |  |
| 10 | My thoughts about the situation makes me feel how I feel. | **0.529** | 0.031 |  |  |
| 11 | My way of thinking, not the situation, is responsible for how I feel | **0.508** | -0.165 |  |  |
| 12 | It is my thoughts about peoples' actions that make me feel how I feel. | **0.469** | 0.150 |  |  |
| 13 | My emotions are caused by my thoughts about events and situations. | **0.457** | 0.069 |  |  |
| 14 | It is my way of thinking that is responsible for my emotions, not the situation | **0.391** | -0.146 |  |  |
| 15 | How I feel is dictated by my thoughts about how people act towards me. | **0.358** | 0.144 |  |  |
| 16 | In order to change how I feel, peoples' actions towards me need to change. | -0.058 | **0.727** | 3.95 | 16.46 |
| 17 | Only removing myself from the situation can alter how I feel | 0.036 | **0.693** |  |  |
| 18 | Only by changing how people act around me can I really change how I feel. | 0.037 | **0.690** |  |  |
| 19 | Only by changing the situation, can I change how I feel. | -0.089 | **0.686** |  |  |
| 20 | I can only change how I feel, by removing myself from the situation. | 0.71 | **0.678** |  |  |
| 21 | To change how I feel, people around me need to change. | 0.038 | **0.627** |  |  |
| 22 | I can change how I feel, only by changing the situation I am in. | -0.081 | **0.618** |  |  |
| 23 | I can change how I feel, only by changing how people act towards me. | -0.002 | **0.585** |  |  |
| 24 | To change how I feel, I need to change the situation. | -0.064 | **0.582** |  |  |

*Notes*. Total variance explained = 42.15%, Model fit: *χ*^2^ = 557.734, df = 229, *p* < .001, Cronbach’s *α* = .78
