# Supplementary material for: Knowing your ABCs: Extending the assessment of stimulus-response (S-R) and cognitive-mediation (C-M) beliefs
Source: PLoS One. 2022 Jun 14;17(6):e0269928. doi: 10.1371/journal.pone.0269928 (PMC9199960; doi:10.1371/journal.pone.0269928)
Supplement: S3 File — (DOCX) [file pone.0269928.s003.docx]

Supplementary file 3. Confirmatory Factor Analysis (CFA) and test of bifactor models.

Following Turner et al. [21], a number of descriptive fit indices were used, following guidelines for goodness of fit indices [49]. Specifically, we used the Root Mean Square Error of Approximation (RMSEA) with a value of less than .08 considered a cut-off for acceptable fit [69]. We also used the Comparative Fit Index (CFI) with a value of .95 indicative of acceptable fit. We also used the Normed Fit Index (NFI) whereby a value of .90 is indicative of acceptable fit [70]. In addition, the Tucker Lewis index (TLI) was used, with values between .90 and .95 considered acceptable (e.g., [69]). We also used the Standardized Root Mean Square Residual (SRMR) whereby a value of below 0.08 is considered acceptable (Kline, 2005). Whilst some criteria of fit may be loosened without causing substantial problems (e.g., CFI/TLI/RNI greater than .90 is acceptable; [67]), we adhered to the Schermelleh-Engel et al. [49] acceptable fit criteria. Modification Indices (MI) values higher than 20 related to sub-factor items were inspected [50], and the covarying of subfactor item errors occurred because some subfactor items possessed similarities in item content [71].

Further, we sought to iteratively remove problematic items using the item factor loadings [49]. Although CFA analysis in the current study is by definition confirmatory, there is still some exploration taking place, with correlated and bifactor models being tested for both two-factor and three-factor models. We wanted to gain a better understanding of the underlying structure of the variables and ensure that we arrived at the appropriate factor structure, and thus did not want to prohibit the possibility of model modification through assessments of factor loadings, and modification indices (MIs) while maintaining the congenericity of the measurement model within the theoretical framework (e.g., [72]). We exercised caution in model modification [73] and in study 3 we recruited a separate sample in order to confirm the factor structure arrived at in study 2, to avoid championing a model that has an artificially good model fit on the basis of a single dataset [74].

To indicate the extent to which the bifactor model offered a better fit to the data than the correlated-factor model, we compared fit indices, and ran *χ*^2^ difference tests. Also, because bifactor models have a propensity to fit any possible data [75], and because even a good fitting model can include misspecification [76], ancillary bifactor measures were used to provide additional information to interpret construct dimensionality [44, 45]. We used: percentage of uncontaminated correlations (PUC; > .70 = unidimensional), explained common variance (ECV; > .70 = unidimensional), OmegaH (> .80 = unidimensional), Relative Omega (higher scores indicate greater unidimentionality), item explained common variance (IECV; > .80 = unidimensional), and Average Relative Measurement Parameter Bias (ARMPB; < 10% is not problematic for unidimentionality).
