# Supplementary material for: Knowing your ABCs: Extending the assessment of stimulus-response (S-R) and cognitive-mediation (C-M) beliefs
Source: PLoS One. 2022 Jun 14;17(6):e0269928. doi: 10.1371/journal.pone.0269928 (PMC9199960; doi:10.1371/journal.pone.0269928)
Supplement: S4 File — (DOCX) [file pone.0269928.s004.docx]

Supplementary file 4. Model fit indices for the correlated and bifactor, two-factor models.

| Model | *χ*^2^ | df | CFI | TLI | NFI | RMSEA (90% CI) | SRMR |
| --- | --- | --- | --- | --- | --- | --- | --- |
| **1. Correlated two-factor** | **114.006** | **62** | **.97** | **.96** | **.93** | **.05 (.036-.065)** | **.046** |
| 2. Bifactor two-factor | 113.723 | 52 | .95 | .92 | .92 | 07 (.055-.084) | .042 |

*Note*. All models were statistically significant at the p < .001 level. χ2 = chi-square test statistic, RMSEA = Root Mean Square Error of Approximation, CI = Confidence Interval, CFI = Comparative Fit Index, TLI = Tucker-Lewis Index, SRMR = Standardised Root Mean Residual. The bolded model demonstrates the best fit in the correlated and bifactor models.
