# Supplementary material for: Knowing your ABCs: Extending the assessment of stimulus-response (S-R) and cognitive-mediation (C-M) beliefs
Source: PLoS One. 2022 Jun 14;17(6):e0269928. doi: 10.1371/journal.pone.0269928 (PMC9199960; doi:10.1371/journal.pone.0269928)
Supplement: S5 File — (DOCX) [file pone.0269928.s005.docx]

Supplementary file 5. Correlated and bifactor two-factor item loadings, Relative Parameter Bias (RPB), and item explained common variances (IECV).

|  |  |  | |  |  | | Bifactor | | |
| --- | --- | --- | --- | --- | --- | --- | --- | --- | --- |
| Item number |  | | Uni factor loading | RPB | EICV | General factor loading | | Specific factor loading |  |
|  | **C-M generation** | |  |  |  |  | |  |  |
| 7 | My thoughts about things around me makes me feel how I feel. | | .145 | 0.832 | .588 | .861 | | .720 |  |
| 3 | How I feel is dictated by my thoughts about the situation. | | .368 | 0.232 | .375 | .479 | | .619 |  |
| 2 | My thoughts about the situation cause me to feel these unpleasant emotions. | | .395 | 0.334 | .411 | .593 | | .710 |  |
| 4 | My emotions are caused by my thoughts about things around me. | | .426 | 0.221 | .368 | .547 | | .717 |  |
| 1 | How I feel is dictated by my thoughts towards things that happen in my life. | | .617 | -0.783 | .246 | .346 | | .605 |  |
| 6 | My emotions are caused by my thoughts about things that happen to me. | | .463 | -0.084 | .303 | .427 | | .647 |  |
| 5 | My thoughts about what happens to me makes me feel these unpleasant emotions. | | .656 | -0.822 | .240 | .360 | | .641 |  |
|  | **S-R change** | |  |  |  |  | |  |  |
| 19 | Only by changing the situation, can I change how I feel. | | .742 | 12.242 | .008 | -.066 | | .737 |  |
| 20 | I can only change how I feel, by removing myself from the situation. | | .724 | -10.677 | .007 | .062 | | .743 |  |
| 18 | Only by changing how people act around me can I really change how I feel. | | .685 | -61.273 | .000 | .011 | | .633 |  |
| 17 | Only removing myself from the situation can alter how I feel | | .802 | 9.717 | .012 | -.092 | | .830 |  |
| 16 | In order to change how I feel, peoples' actions towards me need to change. | | .587 | -7.041 | .020 | .073 | | .516 |  |
| 22 | I can change how I feel, only by changing the situation I am in. | | .556 | 5.413 | .049 | .045 | | .683 |  |
